# Co-creation in healthcare and research to improve service delivery for young people with chronic pain

**DOI:** 10.3389/fmed.2024.1431155

**Published:** 2024-09-12

**Authors:** Carolyn Berryman, Tegan Starr, Nicki Ferencz, Rachael Coakley

**Affiliations:** ^1^Innovation, Implementation and Clinical Translation (IIMPACT) in Health, Allied Health and Human Performance Unit, University of South Australia, Adelaide, SA, Australia; ^2^Brain Stimulation, Imaging and Cognition Research Group, School of Biomedicine, University of Adelaide, Adelaide, SA, Australia; ^3^Hopwood Centre for Neurobiology, South Australian Health and Medical Research Institute, Adelaide, SA, Australia; ^4^Women’s and Children’s Hospital, Adelaide, SA, Australia; ^5^Boston Children’s Hospital and Harvard Medical School, Boston, MA, United States

**Keywords:** pediatric, chronic pain, multidisciplinary, co-creation, collaborative knowledge

## Abstract

**Introduction:**

The process of co-creation can enable more effective, agile and integrated healthcare solutions achieving outcomes that effectively translate to healthcare delivery. Collaborative knowledge generation is particularly important in fields such as pediatric chronic pain where there is a complex interplay between biological, social, environmental, emotional, familial and school factors. The co-creation initiative described here was designed to amplify the voices of youth with chronic pain and their families and a variety of key stakeholders and generate novel approaches to the management of chronic pediatric pain in the setting of the South Australian Pediatric Chronic Pain Service.

**Methods:**

Stakeholders who were identified as influential in this ecosystem were allocated to 6 groups. A skilled facilitator co-prepared and delivered the workshop, engaging participants in three structured activities. Firstly, the challenges to service delivery were outlined, followed by the groups discussing what is currently working. The second activity involved lateral thinking without restrictions on time, resources or system to generate solutions to the key challenges presented. Finally, stakeholders were asked to agree on a generated solution from Activity 2 and build a case for actionable implementation of this solution. Data were summarised by the workshop facilitator and reflexive thematic analysis was used for coding and generating themes.

**Results:**

From Activity 1, six themes collectively demonstrated that stakeholders valued many of the existing strengths of the service delivery, but some areas such as pain education was undervalued. Activity 2 generated solutions from high-level ideas to more day-to- day management strategies. Each of six groups generated unique solutions to an identified challenge for Activity 3.

**Discussion:**

Engaging a wide variety of stakeholders in collaborative knowledge generation successfully provided the South Australian Pediatric Chronic Pain Service with a variety of novel, scalable solution across the healthcare continuum. Equally important is that this initiative helped to raise awareness about the complex issues faced in pediatric chronic pain care and helped to establish new partnerships that have led to enhanced service delivery.

## 1 Introduction

The process of co-creation can enable more effective, agile and integrated healthcare solutions achieving outcomes that effectively translate to healthcare delivery (1, 2). Co-creation supports the optimization of person-centered care through purposeful engagement at all levels of the healthcare and wellness system using facilitated processes and enriched experiences to co-design new services (1, 3). This includes collaborative knowledge generation with consumers, clinicians, academics, government, and policy makers to share insights that align research with consumer needs and service development with improved outcomes.

Collaborative knowledge generation is particularly important in fields such as pediatric chronic pain where there is a complex interplay between biological (4, 5), social (6), environmental (7), emotional (8, 9), familial (10, 11) and school factors (12). Chronic pain is defined as persistent or recurring pain of any cause lasting longer than 3 months (13, 14), and is a common problem in pediatrics with a recent systematic review representing data from 73 countries, placing overall population prevalence at 20.8% (15). Pediatric chronic pain places a considerable financial burden on society. Although not well defined, the total annual costs to society per adolescent with moderate to severe chronic pain in the US was estimated between $7,000 and $12,000 US (16, 17). Data from the Population Health Survey in 2018 (18) can be extrapolated to suggest that 72,000 children are managing chronic pain in South Australia, at a conservative cost of AUD$684 million to society annually (19).

The Pediatric Chronic Pain Service (PCPS) opened in South Australia in March 2018. The service is located in the Women’s and Children’s Hospital, Adelaide, and is the only publicly available service for youth with chronic pain in the state of South Australia. At the 2021 census the total population of South Australia was approximately 1.8 million people and of that number 23% (402,293) of people were between the ages of 0–19 years. The majority of the population live in Adelaide (80%), and 20% in lower density country towns or on farming properties at a distance from Adelaide. Nine percent of households are in regions of relative socio-economic disadvantage (20, 21). Ethnically, South Australians identify as English, Scottish or Irish (54%), Australian (32.5%), German (7.6%), Indian (2.5%) and Aboriginal or Torres Strait Islanders (2.4%) (20).

The PCPS is a multidisciplinary service that delivers a biopsychosocial model of assessment and management of chronic pain (22–26). Youth who attend the service are predominately female (71%), have an average age 13 years, and the most common reasons for referral are abdominal pain (23%), back pain (21%) or daily headache (12%). Thirteen percent of youth report taking daily opioids on presentation. The service team collaborates with young people (aged 0–18 years) with chronic pain, and their families and carers, including wider community outreach into schools if appropriate, to assess physical, psychological, medical and sociocultural factors that contribute to the pain experience. The aim is to provide a combination of targeted and coordinated multidisciplinary interventions inclusive of physical, cognitive behavioral, and medical therapies in the sociocultural context of the youth and family (27–29). This multidisciplinary model of care is the gold standard of care in the complex landscape of young people with chronic pain and meets the guidelines of the World Health Organization (19, 30, 31). Yet, the requirement for service delivery to be of high value, sensitive to the needs of culturally and linguistically diverse community and efficient is challenging because at the time of the workshop, and despite its mandate to service the state, the service was only funded to a total 1.7 full time equivalent positions spread between administration, medical and allied health staff. The co-creation workshop was focused on generating innovative ways this tertiary level of care could deliver effective care more efficiently and with broader reach.

With complexity of care, high prevalence rates and high healthcare costs, pediatric chronic pain is a formidable healthcare problem requiring thoughtful solutions that can work within the constraints of a state-funded healthcare system while optimizing care for patients and carers. The co-creation initiative described here was designed to amplify the voices of youth with chronic pain and their families and a variety of key stakeholders and generate novel approaches to the management of chronic pediatric pain within the context of the Pediatric Chronic Pain Service (PCPS) offered by the Women’s and Children’s Hospital, Adelaide, South Australia.

## 2 Materials and methods

To generate ideas about how to deliver evidence-based and efficient care equitably across a large catchment area, the South Australia PCPS in conjunction with the Robinson Research Institute (The University of Adelaide) facilitated a co-creation workshop. International, national, and local stakeholders were invited to attend (see Table 1 for a breakdown of invited stakeholders) and consent to use the de-identified data was obtained at sign-in. Ethics approval to use the data was received from the Women’s and Children’s Health Network Human Research Ethics Committee 2020/HRE01639.

**TABLE 1:** The numbers of those in invited to and those who participated in the co-creation workshop by sex and role.

Stakeholders invited by role	Gender		Number invited	Number attended	Percent yield
	**F**	**M**			
Acute care nurse	2		2	0	0%
Advocacy representative	1	1	2	1	50%
Anesthetist		2	2	0	0%
Business analyst		1	1	1	100%
Educator	2		2	0	0%
General practitioner	1		1	1	100%
Medical consultant (palliative care/pain/physician)	5	1	6	3	50%
Medical director		2	2	0	0%
Medical registrar	1		1	1	100%
Neonatologist		1	1	0	0%
Parent/carer of youth with chronic pain	3		3	3	100%
Pediatrician	1	2	3	0	0%
Pharmacist	2	1	3	2	67%
Physical therapist	5		5	3	60%
Policy maker	2	1	3	3	100%
Psychologist	9		9	5	55%
Researcher - clinical	2		2	2	100%
Researcher – academic	3	4	7	6	86%
Rural primary health care network	1		1	0	0%
Youth with chronic pain	3		3	3	100%
Total			59	34	

### 2.1 Participants

Fifty-nine stakeholders from diverse fields were identified as influential in this ecosystem and invited to the workshop with the aim of attracting a minimum of 20 participants.

### 2.2 Description of the initiative

Attendees were pre-allocated to 6 stakeholder groups of approximately 6–8 people per group with a purposive mix of different stakeholders and experiences. To address the possibility of power imbalances that might arise from mixing professionals with lived experience participants (particularly youth) we did three things: 1. We employed a skilled facilitator (Dr. Seanna Davidson)^1^ to co-prepare and deliver the workshop explicitly engendering the values of respect, equality and inclusiveness throughout the process. 2. We talked with the youth and parents/carers ahead of the session to let them know what to expect and to let them ask questions. 3. We were purposeful in the allocation of stakeholders to tables, and assigned a buddy at the table to each youth/parent dyad. Each group engaged in three structured activities, facilitated by Dr. Davidson. The workshop was not recorded.

### 2.3 Data synthesis

The workshop facilitator undertook the first synthesis of the data, creating a summary of the generated information. For the first activity we used reflexive theme analysis approach following the example of Braun and Clark (32). NF, CB and TS adopted a constructionist and predominately inductive approach to coding and generating themes.

#### 2.3.1 Activity 1: What works and identifying systems-level challenges

This activity began with a presentation from the PCPS Service Lead that included a brief history about the PCPS service, its current model of multidisciplinary care, patient demographics, service provision, and resources. The PCPS Service Lead also provided a detailed description of seven key challenges that impede service delivery in the PCPS, highlighting how each challenge directly impacts patient care. Challenges included:

1. Insufficient staffing: The PCPS could only support 1 day of new clinic assessments per month and waitlists were 12 months long.
2. Clinical complexity: Young (0–18 years) people with chronic pain and their families require a co-ordinated, flexible and dynamic multidisciplinary approach to care that requires time and resources above ordinary outpatient clinics.
3. Limited accessibility: Indigenous and people who live in rural areas could not access PCPS services easily.
4. Lack of prevalence data: It is very difficult to determine the prevalence of young people with chronic pain (due to variable study populations, heterogenous pain issues, and the absence of centralized data collection across primary and tertiary services), and as such the state-wide unmet need in this area of pediatrics has not been clearly identified.
5. Minimal education for allied health professionals: Pediatric patients with chronic pain often have comprehensive care plans, but most community providers do not have the skills needed to implement these plans, making it difficult to refer patients into the community.
6. Researchers and providers are in silos: The lack of connectivity between clinicians and researchers impedes opportunities to innovate and improve care.
7. Disjointed advocacy: Chronic pain is a widely reported symptoms across illness, injury, and disease populations resulting in disjointed advocacy for better care across a range of clinical settings and policy groups.

Following this presentation, stakeholder groups were first prompted to discuss the key strengths of the service and create a list of “What is working.” Ideas generated were written onto sticky notes and placed on butcher’s paper and each idea represented a unit of analysis. They were then asked to consider how the seven key challenges impacted the current practices in the PCPS from a clinical and resource perspective.

This discussion was followed by a presentation from Associate Professor Rachael Coakley from the Boston Children’s Hospital who spoke about the widely implemented Comfort Ability^®^ Program (CAP) (33). Briefly, CAP is a structured, cognitive-behavioral based workshop offered to adolescents with chronic pain and their carers which may be run over a day or virtually over several weeks. The program has demonstrated outcomes of enhanced pain self-efficacy and functional ability for adolescents and changes in parental beliefs about the ability of their adolescents to manage pain (33) and also may reduce associated maladaptive carer practices that are known to delay recovery (34). Notably, CAP was designed for knowledge and clinical service mobilization with the intent of broadly supporting resource-challenged pain services (such as the PCPS) through shared clinical innovations and a network of providers.

After the presentation, each group was given the opportunity to discuss CAP, considering specifically how adoption of a clinical innovation like CAP could enhance the mission of PCPS and increase access to multidisciplinary chronic pain care in South Australia. Additionally, groups were asked to discuss which of the seven key challenges CAP would help to address.

#### 2.3.2 Activity 2: Linking challenges to solutions

In a second activity the same stakeholder groups were asked to “think outside the box” and without restrictions on time, resources or system, generate solutions to the seven key challenges set out by the PCPS. The goal was to elicit a range of perspectives and approaches for solving the complex challenges faced by the PCPS. Given the diversity of stakeholders, solutions represented a variety of high-level ideas (i.e., changes in government policy) as well as more day-to-day management strategies (i.e., changing the frequency of clinic schedules). These ideas were generated collaboratively and recorded by group. Each idea represented a unit of analysis. We determined the importance of the challenges (which ones need addressing first) by the percentage of responses provided for each challenge.

#### 2.3.3 Activity 3: Refining the South Australian model

In the third activity, each stakeholder group was asked to agree on a generated solution that best fit with the vision of the program, “*an interdisciplinary service that offers a coordinated, evidence-based, therapeutic intervention for the effective treatment of persistent pain*” and that was achievable, given the significant challenges faced by the PCPS. Once this solution was selected, they were asked to build a case for actionable implementation of this solution by responding to the following four questions:

1. What challenge is this solution addressing?
2. What is the action/change that is needed for this solution?
3. How might this solution be made feasible for PCPS?
4. What impact would this solution have if implemented?

The workshop concluded with each stakeholder group presenting their actionable solutions to the broader group and discussing imperative next steps to support the growth and development of the PCPS. Stakeholders were united in their recognition of the current strength of the PCPS service as well as the need for immediate clinical expansion and long-term solutions for sustainability.

## 3 Results

In total 34 stakeholders attended the workshop (see Table 1), a clear indication of the strong interest in improving pediatric pain care in South Australia.

Qualitative data and feedback was generated collaboratively, collected during this workshop, and collated. Data management procedures and outcomes are described by each activity below. The full data set can be accessed:

### 3.1 Activity 1: What works and identifying systems-level challenges

Each stakeholder’s response to the question “what is working?” was considered a unit of analysis. On average, each stakeholder provided three responses and in total 175 responses were collected. Three authors independently reviewed the data and used an inductive approach to classify responses into themes, agreeing on a total of six themes. The number of responses per theme and an example of the responses can be seen in Table 2.

**TABLE 2:** Responses to “What is working [within the service]?” organized into six themes and presented by the number of responses by participant per theme.

Themes	Percent response (n)	Description of activities within the themes
Multidisciplinary model of care	32% (56)	• Investment in allied health • Active approaches • Patient and family centered treatment • Skilled use of language, presenting choices, deep listening and validation • Bring patients together to hear from each other, decrease isolation and increase support • Marrying the concept of child development stages to treatment choices
Collaborative care/advocacy	31% (54)	• Widespread community support and awareness • Clear and accessible pathways and links between primary and tertiary care, acute and chronic pain teams, child to adult care • Supporting partnership with family doctors • University, industry and consumer partnerships • Lobby to put pediatric chronic pain on the government radar
Multi-level whole of community pain education strategies	17% (29)	• Involve the whole family in pain education • Upskill peer supporters/mentors • Generate a bank of success stories for sharing • Use stories and metaphors to explain chronic pain and the mind-body connection • Shared language of pain and consistent messaging about pain • Talking about pain in the medical community
Data support and funding	9% (16)	• Data collection to support service development • National data collection initiative can be used to benchmark service • Collecting and sharing data to identify patterns • Factor in sustainability • National and international collaborations to secure funding • Use data to lobby for funding and underpin grant success
Research/clinical science	8% (14)	• Value the importance of evaluating new initiatives/strategies • Youth with chronic pain and their parents/carers integrated into research • Opioids don’t have a role in chronic pain • Evidence based interventions tailored to condition • National and international collaborations to integrate clinical care and research • Links with Universities to translate research into education and practice • Evaluated tools, manuals and workbooks that are iterative • Provide a variety of evidence-based ideas for service development
Use of technology and telehealth	3% (6)	• Telehealth—improve rural and equitable access • Virtual reality • APPs such as period tracking apps, day management apps and pain apps

The higher the number of responses, the stronger the theme is perceived to be working within the service.

The six themes collectively demonstrate that stakeholders valued many of the strengths of existing practices of PCPS and have a working knowledge of the complex treatment and management of pediatric chronic pain. The first two themes (i.e., importance of multidisciplinary model of care and community and collaborative care) reflected the key strengths of the service, providing best practice care according to guidelines for clinical management of chronic pain in children from the World Health Organization (19). These guidelines encourage treatment of chronic pain from a biopsychosocial and interdisciplinary model. As pediatric chronic pain not only impacts the child, but also the social fabric of the child’s life, it is imperative that any approaches align with family, school, leisure time and economic considerations [World Health Organization (19), p. 15–16].

However, there were also places where stakeholders may have undervalued the strengths of essential components of the PCPS. For example, only 17% of stakeholders placed value on pain education for patients and providers. This may in part be due to the lack of pain education in undergraduate medical and allied health programs (35, 36) and in part because many stakeholders have not previously been exposed to this essential area of training in pediatric practice. The final three themes presented opportunities to build strength in the areas of data support, funding, research and use of technology.

### 3.2 Activity 2: Linking challenges to solutions

Data evaluation for Activity 2 similarly included categorization of stakeholder responses. In this activity stakeholder groups identified solutions for specific PCPS challenges. Ninety responses were received and data were categorized by the challenge they address (see Table 3). Given the diversity of stakeholders, solutions represented a variety of high-level ideas (i.e., lobby for changes in University healthcare education) as well as more day-to-day management strategies (i.e., use readily available resources). Unique responses accounted for many of the solutions, showing the strength of bringing together such a diverse group. The top three challenges for the service were 1. Insufficient staffing, 2. Clinical complexity, 3. Minimal pain education for all healthcare professionals.

**TABLE 3:** Linking challenges to solutions and actionable steps.

PCPS key challenges	Percent responses (n)	Sample stakeholder solutions and actionable steps
1. Insufficient staffing	21% (19)	• Develop relationships with universities to take students on clinical placement • Upskill the parent/carer with pain management strategies • Include disciplines currently under-utilized such as pharmacy and the families’ General Practitioner • Use readily available internet resources for chronic pain and other published material to enhance communication and learning • Establish group programs
2. Clinical complexity	17% (16)	• Develop a shared language around pain • Move the focus of treatment toward promoting comfort and function and away from medication • Focus efforts on improving communication between healthcare professionals, families and school and social supports embedded in the interdisciplinary approach
3. Limited accessibility	13% (12)	• Enhance online opportunities for education through credible resources already available (e.g., online telehealth consultations and group programs or chats) • Conduct outreach via school programs for pain • Contact universities to determine whether there are existing outreach programs for rural communities where pain could be an “add-on” to current care models
4. Lack of prevalence data	8% (8)	• Use other countries such as Canada and the United States of America as a blueprint for service development of a state-wide pain clinic • Share information regarding prevalence more broadly • Create an advisory board to assist with the more complex decisions around meeting system needs • Develop a prevalence study to guide service development
5. Minimal pain education for all health professionals	17% (15)	• Lobby for improvements in university level pain education • Provide outreach education and develop an intervention that extends into the community • Build capacity amongst patients/peer mentors and practitioners • Incorporate pain education for all healthcare practitioners into Hospital Grand rounds and inhouse education • Coordinate access to community allied healthcare providers through GP care planning • Collaborate with education committees in tertiary healthcare institutions to develop a common understanding and capacity to manage chronic pain
6. Researchers and providers are in silos	10% (9)	• Foster research within the service by embedding student led research • Build a research translation culture by disseminating relevant research to service providers • Enhance efforts to move evidence-based care models into practice
7. Disjointed advocacy	12% (11)	• Learn how to advocate, develop patient advocates, and lobby for chronic pain in children to be recognized at the state policy level • Use service data collection to support the development of the service and promote early intervention • Use client and community engagement to promote advocacy • Establish a closed moderated private social media group for children and young adults with chronic pain to share their journey with peers.

### 3.3 Activity 3: Refining the South Australian model

Data for Activity 3 included a brief written summary of ideas to refine the PCPS model of care in South Australia (SA). This involved stakeholder groups selecting a key problem, proposing solutions, and identifying and actionable steps for implementation which have been integrated into the solutions linked to challenges in Activity 2 (see Table 3). In total there were six groups of stakeholders and each group generated unique solutions to an identified challenge. Additionally, this activity generated vibrant discussion within and across stakeholder groups. The identified challenges set forth by each group established that stakeholders had a clear understanding of the complexity of the issues faced by the PCPS. The proposed solutions were well aligned with the challenges and again reflected the unique perspectives of the heterogenous stakeholder groups and provided suggestions for directions for the service to pursue in the future.

## 4 Discussion

Engaging a wide variety of stakeholders in a collaborative problem-solving workshop such as this successfully provided the PCPS with a variety of novel ideas and solutions across the healthcare continuum. Equally as important, this initiative helped to raise awareness about the complex issues faced in pediatric chronic pain care and helped to establish new partnerships that have led to enhanced service delivery. Stakeholders promoted systems level change in medical education, government policy, hospital administration, community, and allied health professional networks. Moreover, suggested improvements in the South Australian model targeted physicians, psychologists, educators and consumers. All stakeholder groups acknowledged the need for comprehensive improvements in pediatric chronic pain care, but also thought realistically about attainable and sustainable solutions for the PCPS and South Australia.

While some stakeholders presented longer-term systems level changes (e.g., implementation of new public healthcare policies), many also identified short-term practical solutions that may help to improve service delivery (e.g., provider webinar to enhance referral pipelines). Most participants within this co-creation workshop concluded that many of the day-to-day clinical challenges faced by the PCPS reflected a need to optimize state-wide management of pediatric chronic pain. This can occur through a variety of state-based initiatives such as increasing quality pain education for health professionals, linking hospital and community services, and enhancing network connections between clinicians, researchers, and consumers.

Encouragingly, stakeholders were energized by this activity and many expressed interests in further engagement with PCPS and improving South Australian pediatric pain care more broadly. Given that comprehensive change requires a network of engaged stakeholders beyond this co-creation workshop, the relationship building that occurred within the context of this co-creation workshop was a highly valued outcome. Importantly, while the PCPS faces formidable challenges, this workshop also served to highlight the many areas in which they are succeeding in their endeavor to provide high-quality, comprehensive pain care to patients and their carers.

Co-creation in healthcare has been applied in the therapeutic situation when co-creation amongst stakeholders is used to successfully focus healthcare professionals on generating meaningful outcomes for the patient (37). Co-created therapeutic decision making goes beyond shared decision making in the clinical setting to include all healthcare and non-healthcare environments that are important to the patient such as social media environments, environments in which patients find pleasure and relationships between family, peers and friends in the decision making process. The patient is at the center of care and also takes care of themselves along a continuum of interactivity with all stakeholders. Practically, co-created decision making aims to improve the patient’s wellbeing by including the patient as an active participant in the process. Co-design has also been used to refine the “GETLiving” program for youth with chronic pain (38). Three co-design meetings with youth with chronic pain and three parallel meetings with parents/carers were run to prioritize 12 ideas for program improvement that had been generated by earlier interviews. Within and between group consensus was used to rank the ideas by importance, providing the organizers of the program with a clear understanding of what key elements of the program were most helpful to participants.

Since the conclusion of this workshop, the PCPS has taken steps toward implementing changes that were first discussed within this workshop. For example, the cognitive behavioral workshop, CAP, has been successfully implemented and is running three times a year. CAP facilitates knowledge about the underlying contributions and impacts of chronic pain in young people and their families and carers and promotes vocabulary to talk about pain, shared experiences, and perspective taking. Consumer feedback from this implementation has been very positive and the PCPS are finalizing the outcomes of a research study that has evaluated the feasibility and acceptability of adapting the workshop to Australian needs. This initiative aligns with the stakeholder’s concern around improved pain education, scalable interventions, and access to care at the community level. Additionally, research engagement and collaborations between researchers and clinicians connected to the CAP network has continued to develop. Indeed, the PCPS has been instrumental in supporting the instantiation of the CAP workshop in Western Australian where it is run both face to face and online and in Queensland.

Quality assurance data from this workshop and stakeholder investment has also helped to support the PCPS in increasing their staffing levels. Increased and stable funding has enabled the service to build up a dedicated multidisciplinary team over three days each week with a view to increasing to five days a week in mid-2024. The service is now permanently established and consistently able to respond to patient referrals within timeframes recommended by the guidelines of the WHO for the management of chronic pain in children (19).

Within the Women’s and Children’s Hospital (WCH) the PCPS has also increased communication and knowledge about pediatric pain. For example, they fostered a joint understanding with the Emergency Department by sharing resources and approaches to pain flares (hypnotherapy, pain education), presented to the wider WCH community at the weekly Grand Round and been in regular communication with the Director of Consumer and Community Engagement, presenting the mission of the PCPS to consumer and community advisory groups and using the feedback from these groups for the development of education resources, research priorities and resources. The PCPS has invited leading clinicians from the pelvic pain treatment service, cancer care service and rheumatology to observe the CAP workshop and encouraged shared care between these services.

Additionally, a culture of student led research has been embedded within the PCPS, fostering closer links with University faculties and enabling clinical outcomes to inform service improvement. Further, collaborations with national researchers and stakeholders have won funding to improve the model of care by codesign, strengthening stakeholder engagement with the service and raising the profile of pediatric chronic pain within central government bodies. The PCPS has become a site for clinical innovation, collaborating with other national and international pain services to trial a custom developed, pain specific virtual reality program to augment interventions targeting pain and pain rehabilitation in pediatric populations.

### 4.1 Strengths and limitations

Competing demands prevented 25 invitees from attending the workshop, but we recruited more than our desired sample size of 20 people (34 attended) from a diverse range of stakeholders. A limitation of our process was that we were not funded for a consensus meeting with participants and were unable to check whether the themes that we generated resonated with them. Finally, strong engagement with current healthcare systems is needed to promote long term sustainability and scale out of the solutions and the success of this will depend on healthcare system funding priorities.

## 5 Conclusion

The South Australian PCPS led a co-creation workshop to bring together key stakeholders including clinicians, researchers, administrators, policy makers, patients, and carers. The primary goal was to educate participants about the strengths and challenges faced by the newly launched PCPS and to generate innovative ideas and solutions to support pediatric chronic pain care in South Australia. Most workshop participants valued existing services for youth with chronic pain. However, there was also widespread recognition that systems-level challenges impede the quality and availability of care.

Since the completion of this stakeholder workshop, PCPS has grown significantly, successfully addressing issues in all seven key challenge areas identified by the service. Specifically, they have improved referral pipelines, increased state funding and full time equivalents (FTE’s), engaged in community education, enhanced advocacy, built new partnerships within and outside WCH, implemented additional evidence-based practices, improved access to care, worked in collaboration with consumers (patients and carers), and developed several collaborative research initiatives with universities across Australia and internationally. It is strongly believed that this co-creation workshop helped to spur this change by generating novel ideas, providing data for PCPS leadership, and establishing new allies who could help to champion growth.

Ongoing challenges include developing safe and regularly convened communities for young people with chronic pain, no matter their linguistic or cultural diversity, to discuss their situations with peers, building healthcare professional capacity to manage chronic pain in young people beyond the PCPS team, and developing models of care coordinated across primary and tertiary centers. To meet these challenges the PCPS will continue to engage a wide variety of stakeholders in a solutions-focused approach to improving pediatric chronic pain care in South Australia.

## Data Availability

The datasets presented in this study can be found at https://doi.org/10.6084/m9.figshare.25800499.v1.

## Footnotes

1
https://www.the-systems-school.org/about
